# Food Waste as a Resource for Functional Food Development: From Recovery and Processing to Food Application

**DOI:** 10.3390/foods15213936

**Published:** 2026-11-03

**Authors:** Gabriele Ballistreri

**Affiliations:** Consiglio per la Ricerca in Agricoltura e l’Analisi dell’Economia Agraria (CREA), Research Center for Olive, Fruit and Citrus Crops, Corso Savoia 190, 95024 Acireale, Italy; gabriele.ballistreri@crea.gov.it

## 1. Introduction

Food production and processing generate heterogeneous side-streams that include peels, seeds, pomaces, cakes, meals, cereal fractions, coffee residues, and dairy co-products. Although these materials are often treated as waste, many retain dietary fiber, proteins, lipids, minerals, and phytochemicals with nutritional, technological, or bioactive potential. Their recovery can reduce disposal burdens while providing new raw materials for food production. However, composition varies with species, cultivar, maturity, processing history, and storage, so the presence of valuable compounds alone does not establish that a side-stream is suitable for use as a food ingredient [1,2]. In this Editorial, the term “side-stream” is used broadly to encompass processing by-products and residual fractions that may still be suitable for valorization; this does not imply that every material currently classified as waste can be returned to the food chain.

Valorization therefore requires a sequence of connected operations. The source material must first be characterized and stabilized; target compounds or functional fractions must then be recovered or generated under food-compatible conditions; and the resulting ingredient must perform reliably in a food matrix. Recovery strategies should also minimize solvent use, energy demand, and degradation of target compounds. These requirements underpin both the established framework for recovering high-value components from food waste and the principles of green extraction [3,4].

A further distinction is needed between a bioactive-rich laboratory extract and a functional food. The latter requires evidence that processing preserves the relevant constituents, that the ingredient is safe and technologically compatible with the product, and that any claimed physiological benefit is supported by appropriate efficacy testing [5]. Sensory quality, shelf stability, regulatory status, and economic feasibility are therefore part of functional food development rather than secondary considerations.

The Special Issue “Food Waste as Ingredients in Functional Food Development: Sources, Extractions and Functionality” brings together four reviews and six research articles addressing this progression. The contributions cover plant- and dairy-derived side-streams, compositional characterization, drying, green extraction, microbial fermentation, and incorporation into formulated foods. Considered together, they show that successful upcycling depends on matching the properties of each matrix with an appropriate processing route and intended food application.

## 2. From Food Side-Streams to Functional Ingredients: Contributions of the Special Issue

Characterization is the first step because side-streams that share a general name may differ substantially in composition and end-use potential. Guardianelli et al. (Contribution 1) compared grape pomace flours from Argentinian Isabella grapes and Italian Cabernet grapes and identified differences in dietary fiber, minerals, fatty acids, individual phenolics, and antioxidant activity. Their findings support the application-specific use of grape pomace rather than its treatment as a uniform ingredient: Cabernet pomace was particularly rich in dietary fiber and several minerals, whereas Isabella pomace contained higher levels of unsaturated fatty acids and markedly higher concentrations of several phenolic compounds, including catechin, epicatechin, caffeic acid derivatives, and anthocyanins. Stabilization can also determine which properties are retained.

Ramírez-Aguirre et al. (Contribution 2) examined convective drying of avocado seeds across different temperatures, sample thicknesses, and air velocities. Their multi-response analysis identified separate processing scenarios for a phytochemical-rich concentrate, a soluble ingredient, and a water-retaining matrix, showing why a single global optimum may be inappropriate when the desired food function differs. The study also showed that a lower drying temperature did not necessarily mean lower estimated energy use because longer treatment times could offset the lower thermal load.

The broader diversity of potential sources is illustrated by two reviews. Nemli et al. (Contribution 3) examined residues from plant-based oil processing, including pomaces, cakes, meals, seeds, peels, and wastewaters. These materials can supply phenolics, lignans, fiber, protein, and minerals, but their use requires suitable recovery methods, effective management of compositional variability, and compliance with safety and regulatory requirements. Pagnotta et al. (Contribution 4) focused on glucosinolate-containing Brassicales species and described how agricultural residues and processing by-products may provide functional ingredients. The review also emphasized the influence of agronomic practices, postharvest handling, processing, and fermentation on glucosinolates and their bioactive hydrolysis products. These contributions reinforce the need to define the ingredient by its composition, process history, and intended function, rather than by its source alone.

Once the raw material has been stabilized, recovery processes must be selected and compared under controlled conditions. Ballistreri et al. (Contribution 5) first optimized the water-to-ethanol ratio for phenolic recovery from lemon processing by-products and then compared conventional extraction, ultrasound-assisted extraction, and hydrodynamic cavitation using the same solvent system. Compared with conventional extraction, ultrasound-assisted extraction increased total flavanone recovery by 25.9% and total polyphenols by 20.5%. Hydrodynamic cavitation provided a further increase of 12.0% in flavanones and 5.2% in total polyphenols relative to ultrasound-assisted extraction under the tested conditions. This work also highlights an important methodological point: conclusions about extraction technologies are more reliable when solvent composition, solid-to-liquid ratio, temperature, and post-extraction treatment are controlled. Ultrasound-assisted extraction and related intensified processes can reduce treatment time and solvent demand, but performance remains matrix- and process-dependent [4,6].

Biological processing provides a complementary route because microorganisms and their enzymes can release bound constituents, transform substrates, and generate new metabolites. Foti et al. (Contribution 6) reviewed the use of fermentation and microbial engineering to produce bioactive molecules from foods and agrifood by-products. They also noted that starter selection, process control, safety, labeling, and regulatory acceptance are essential when these approaches move toward industrial use. Guzińska et al. (Contribution 7) provided an experimental example using roasted coffee silverskin. Fermentation with a consortium of yeast, acetic acid bacteria, and lactic acid bacteria increased total phenolic content and improved antioxidant capacity by approximately 28% in the ABTS assay and 20% in the DPPH assay. The fermented material also supported probiotic viability under simulated intestinal conditions. These findings justify further work, but they do not yet demonstrate a prebiotic or health effect in vivo; the authors accordingly identified biological validation, sensory testing, and assessment in real food matrices as necessary next steps. Selmi et al. (Contribution 8) extended the bioprocessing perspective to whey, a dairy side-stream that differs markedly from lignocellulosic plant residues. Their review described fractionation and fermentation routes for recovering whey proteins, peptides, lactose-derived compounds, organic acids, polymers, and other products, while identifying palatability, demineralization, safety control, and techno-economic and life-cycle evaluation as continuing challenges.

The final test of valorization is the performance of an ingredient in a food product. Sánchez-Nuño et al. (Contribution 9) incorporated carrot bagasse flour, blue corn flour, and ellagic acid into an extruded snack and optimized die temperature and screw speed based on physicochemical, antioxidant, and sensory responses. The selected operating conditions of 144 °C and 207 rpm produced an acceptable combination of expansion, texture, water-related properties, antioxidant capacity, and sensory response. The study demonstrates why formulation and processing must be optimized together, while any health-related effects of the product would still require direct biological and clinical evidence. Săpoi et al. (Contribution 10) developed myrobalan fruit leathers enriched with blackcurrant or bilberry pomace powders. Pomace addition improved phenolic content, antioxidant activity, color, taste, and flavor, while the work also showed how sugar level influenced texture, acidity, color, and antioxidant properties. Bilberry pomace produced the larger increase in total phenolic content under the tested formulations. Larger sensory panels, packaging and storage studies, and economic and sustainability assessments remain necessary before commercial translation. Together, these food-application studies show that compositional enrichment is meaningful only when the product also satisfies processing and sensory requirements.

## 3. Remaining Challenges and Future Perspectives

The contributions identify several priorities for the next stage of research. The first is standardization. Seasonal, geographical, varietal, and processing differences can alter the composition of side-streams, as illustrated by the contrasting grape pomaces in Contribution 1. Future studies should define acceptance criteria for raw materials, clearly indicate whether results are expressed on a wet- or dry-matter basis, and document storage and stabilization conditions. Marker compounds, compositional ranges, and techno-functional specifications will be needed if experimental powders or extracts are to become reproducible food ingredients.

A second priority is process comparison at a scale relevant to manufacturing. High extraction yield does not by itself establish sustainability. Solvent recovery, water and energy consumption, throughput, equipment requirements, waste generated after extraction, and the stability of the recovered fraction all influence the environmental and economic outcome. The pilot-scale results obtained with hydrodynamic cavitation in Contribution 5 are promising, but they also illustrate why scale-up validation, techno-economic assessment, and life-cycle assessment should accompany further process development. Similar considerations apply to drying, fractionation, membrane operations, and fermentation. Reporting energy and mass balances would make comparisons across technologies more informative.

Safety and regulatory suitability require equally early attention. Food side-streams may concentrate pesticide residues, mycotoxins, environmental contaminants, anti-nutritional factors, allergens, or microbial hazards. Their profiles also depend on the upstream process and storage conditions. A source may therefore be chemically valuable but unsuitable for food use without defined controls [7]. Research should integrate contaminant screening, microbiological criteria, traceability, stability testing, and an assessment of the regulatory status of the resulting ingredient. These issues are particularly important when fermentation changes matrix composition, when novel microorganisms are used, or when an ingredient has no established history of consumption.

Evidence of functionality must also progress beyond compositional analysis and chemical antioxidant assays. Total phenolic content, DPPH, ABTS, FRAP, and ORAC assays are useful for screening and process comparison, but they do not demonstrate bioavailability or a health effect in humans. Simulated digestion, intestinal transport models, targeted mechanistic studies, animal studies, where justified, and well-designed human trials should be selected according to the proposed claim. Functional food development also requires realistic serving levels and evaluation of interactions with the food matrix. This staged approach is consistent with the need to link product design, efficacy testing, and safety assessment [5].

Finally, technological and consumer performance must be evaluated in real foods. Incorporation can change color, flavor, texture, water activity, oxidation, microbial stability, and shelf life. The two formulated foods in Contributions 9 and 10 demonstrate the value of sensory assessment, but broader consumer testing and storage studies are needed before market readiness can be inferred. Collaboration among food chemists, microbiologists, process engineers, nutrition scientists, sensory scientists, and industry partners will be essential. The central research question is no longer whether food side-streams contain valuable compounds, but whether they can be converted consistently into safe, stable, acceptable, and economically viable ingredients.

## 4. Conclusions

This Special Issue presents the valorization of food side-streams as an integrated pathway rather than as a single recovery step. Its contributions encompass compositional characterization, stabilization, extraction, microbial transformation, ingredient development, and food formulation. Collectively, they demonstrate that the most appropriate valorization strategy depends on both the characteristics of the starting matrix and the intended function of the final ingredient.

Further progress will depend on standardized raw material specifications, food-grade and resource-efficient processing, safety and regulatory assessment, evidence of bioaccessibility, bioavailability, and biological efficacy, and on validation in real food systems. Scale-up should be supported by mass and energy balances, techno-economic analysis, and life-cycle assessment. By integrating these elements from the outset of product development, side-streams from food processing can be transformed from promising laboratory-scale materials into reliable functional ingredients, thereby supporting innovation in functional foods and the transition toward a more circular food system.

## Data Availability

No new data were created or analyzed in this study. Data sharing is not applicable to this article.
